# Ultrasound-Promoted One-Pot, Four-Component Synthesis of Pyridin-2(*1H*)-One Derivatives

**DOI:** 10.3390/molecules181214519

**Published:** 2013-11-25

**Authors:** Jinming Yang, Qiang Li, Juanjuan Zhang, Wei Lin, Juxian Wang, Yucheng Wang, Zhibin Huang, Daqing Shi

**Affiliations:** 1 School of Pharmacy, Yancheng Teachers University, Yancheng 224051, Jiangsu, China; 2 Key Laboratory of Organic Synthesis of Jiangsu Province, College of Chemistry, Chemical Engineering and Materials Science, Soochow University, Suzhou 215123, Jiangsu, China; 3 Hainan Chuntch Pharmaceutical Company Limited, Haikou 570216, Hainan, China; 4 Institute of Medicinal Biotechnology, Chinese Academy of Medical Sciences and Perkin Union, Medical College, Beijing 100050, China

**Keywords:** pyridin-2(*1H*)-one, ultrasound irradiation, multicomponent reaction

## Abstract

An efficient one-pot synthesis of 1,6-diamino-2-oxo-1,2,3,4-tetrahydro- pyridine-3,5-dicarbonitrile derivatives by four-component piperidine-catalyzed reactions of a ketone, malononitrile, ethyl cyanoacetate and hydrazine hydrate under ultrasound irradiation is described. This method provides several advantages such as shorter reaction times, excellent yields, and a simple workup procedure.

## 1. Introduction

Multi-component reactions (MCRs) have been designed to produce elaborate biologically active compounds and have become an important area of research in organic, combinatorial, and medicinal chemistry [1,2,3,4]. MCRs offer a wide range of possibilities for the efficient construction of highly complex molecules in a single procedure, thus avoiding the complicated purification operations and allowing savings of both solvents and reagents, making them perfectly amenable to automation for combinatorial synthesis. In the past decade there have been tremendous developments in MCRs and great efforts continue to be made to develop new MCRs [5,6,7,8,9].

Nitrogen-containing heterocyclic compounds are widespread in natural products and medicinal agents [10], and their applications in biologically active pharmaceuticals, agrochemicals, and functional materials are becoming more and more important [11,12]. Among them, pyridinone derivatives have been received considerable attention as a result of their biological activities and as an interesting template for medicinal chemistry [13,14,15,16]. The conventional method for the synthesis of pyridinones is ammonization of pyranone at a high temperature or in a sealed tube [17,18]. Recently, a number of improved methods have been reported in the literatures for the synthesis of this heterocyclic system [19,20,21]. However, most of these methodologies suffer from disadvantages such as multi-step procedures, long reaction times, unsatisfactory yields, and the use of organic solvents or toxic reagents. These facts prompted us towards further investigation in search for a more efficient methods for the preparation of this kind of compounds.

Ultrasound irradiation has been increasingly used in organic synthesis in recent years. A large number of organic reactions can be carried out in a higher yield, shorter reaction time and under milder reaction conditions under ultrasonication. Compared with traditional methods, this method is more convenient and can be easily controlled [22]. Nevertheless, the use of ultrasound in heterocyclic systems has not been fully explored [23,24]. As a consequence of our interest in the synthesis of heterocyclic compounds under ultrasound irradiation [25,26,27,28,29,30], we report herein for the first time a facile one-pot synthesis of pyridin-2(*1H*)-one derivatives via four-component reactions of a ketone, malononitrile, ethyl cyanoacetate and hydrazine catalyzed by piperidine under ultrasound irradiation.

## 2. Results and Discussion

**Scheme 1:**
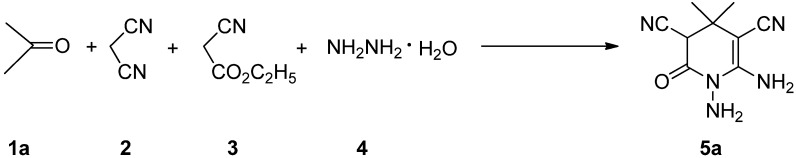
The model reaction.

Initially, the four-component reaction of acetone (**1a**), malononitrile (**2**), ethyl cyanoacetate (**3**) and hydrazine hydrate (**4**) as a simple model was investigated to establish the feasibility of the strategy and to optimize the reaction conditions (Scheme 1). The effects of solvents and catalysts were evaluated for this model reaction, and the results are summarized in Table 1. It was found that when the reaction was carried out without any catalysts only traces of product were detected, even after 10 h under ultrasound irradiation (Table 1, entry 1). To improve the yields, we examined this reaction using different bases (Table 1, entries 2–6). Based on the reaction times and the yields, piperidine was identified as the optimal catalyst with **5a** being isolated in 93% yield (Table 1, entry 2). In order to further improve the product yields, we tried to perform the reaction in higher temperatures under ultrasound irradiation, but the yield did not increase (Table 1, entries 7–8). Subsequently, we turned to testing the effect of solvents. MeOH, CH_3_CN, THF, and water showed no superiority to EtOH (Table 1, entries 9–12). Therefore, EtOH is the solvent of choice for this reaction. To optimize the catalyst loading, 5, 10, 15, 20 and 25 mol% of piperidine was tested, respectively (Table 1, entries 13, 2, 14–16). A 10 mol% loading of piperidine was sufficient to push the reaction forward and 5 mol% of piperidine was not enough. Higher amounts of piperidine did not lead to a significant changes in the reaction yields.

**Table 1:** Optimization of reaction conditions ^a^.

Entry	Solvent	Temperature (°C)	Catalyst	Time (min)	Isolated Yield (%)
1	EtOH	rt	No catalyst	600	trace
2	EtOH	rt	Piperidine (10%)	30	93
3	EtOH	rt	NaOH (10%)	240	80
4	EtOH	rt	KOH (10%)	180	78
5	EtOH	rt	Na_2_CO_3_ (10%)	360	62
6	EtOH	rt	EtONa (10%)	240	83
7	EtOH	40	Piperidine (10%)	30	89
8	EtOH	50	Piperidine (10%)	30	85
9	MeOH	rt	Piperidine (10%)	40	85
10	CH_3_CN	rt	Piperidine (10%)	120	65
11	THF	rt	Piperidine (10%)	90	79
12	Water	rt	Piperidine (10%)	120	trace
13	EtOH	rt	Piperidine (5%)	60	86
14	EtOH	rt	Piperidine (15%)	60	90
15	EtOH	rt	Piperidine (20%)	30	91
16	EtOH	rt	Piperidine (25%)	30	89

^a ^*Reaction conditions*: acetone (1 nmol), malononitrile (1 nmol), ethyl cyanoacetate (1 nmol), hydrazine hydrate (1 nmol) and piperidine (0.1 nmol) in solvent (10 mL) under ultrasonic waves and the ultrasonic power 250 W, irradiation frequency 40 kHz.

**Scheme 2:**
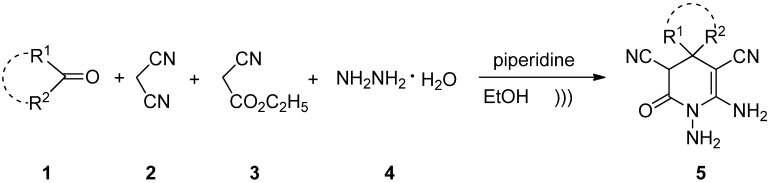
The synthesis of pyridin-2(*1H*)-one derivatives **5**.

Using the optimal conditions, we investigated the substrate scope of the transformation (Scheme 2). The results are summarized in Table 2. As shown in Table 2, aliphatic chain ketones and cyclic ketones were well tolerated under the reaction conditions, leading to the final products in satisfactory yields. However, when the aromatic ketones (such as acetophenone, 4-bromoacetophenone, and 4-methylacetophenone) and aromatic aldehyde (such as benzaldehyde) were used, only traces of products were detected.

**Table 2:** Synthesis of pyridin-2(*1H*)-one derivatives **5**.

Entry	Ketone	Product	With US		Without US	
			Time (min)	Yield (%)	Time (min)	Yield (%)
1	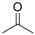	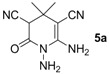	30	94	120	72
2	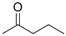	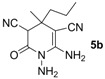	30	92	120	69
3	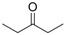	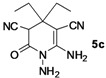	30	93	180	70
4	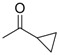	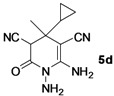	30	92	180	60
5	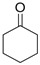	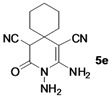	35	92	180	65
6	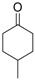	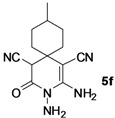	40	91	180	67
7	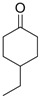	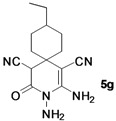	40	90	180	62
8	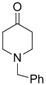	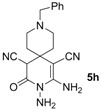	40	91	240	59
9	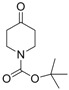	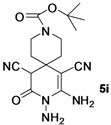	40	89	300	60
10	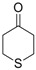	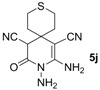	35	88	300	60
11	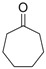	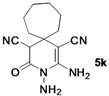	35	89	180	64

**Figure 1:**
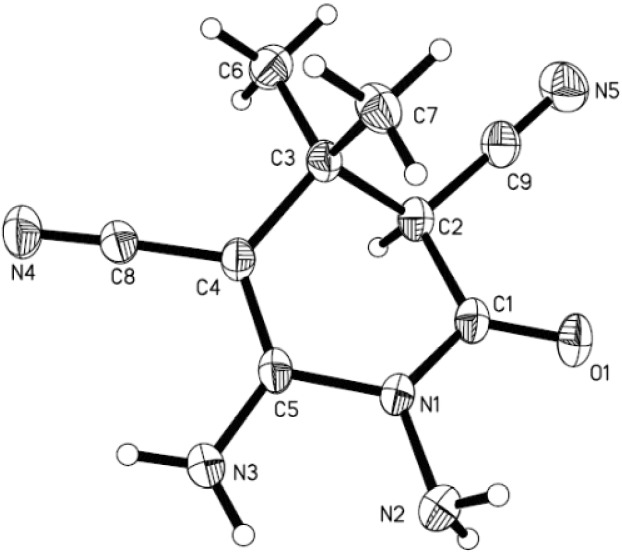
The crystal structure of compound **5a**.

The structures of **5** were characterized using IR, ^1^H-NMR, and ^13^C-NMR spectroscopies, and HRMS analysis. Compound **5a** exhibited characteristic IR stretching frequencies in the 3415, 3346, 3305, 2175, 1702, and 1632 cm^−1^ regions for NH_2_, CN, C=O, and C=C, respectively. In the ^1^H-NMR spectrum of compound **5a** the amino group protons show two singlets at δ 6.61 and 5.15. The methyl group protons show two singlets at δ 1.20 and 1.04 due to the two methyl groups. A singlet appearing at δ 4.50 was assigned to the C-3 proton of the pyridine ring. In addition, HRMS analyses were consistent with the structures. The structure of **5a** was further confirmed by X-ray diffraction. The molecular structure of **5a** is shown in Figure 1.

In this tetrahydropyridine ring, because of the existence of conjugation, the distance C4–N4 [1.338(3) Å] is significantly shorter than the typical Csp^2^–N bond distance (1.426 Å) [31]. The tetrahydropyridine ring adopts a distorted boat conformation, atoms C3, N1, C4 and C5 are coplanar, while C1 and C2 deviate from the plane by 0.4358(39) and −0.3639(38) Å, respectively.

**Scheme 3:**
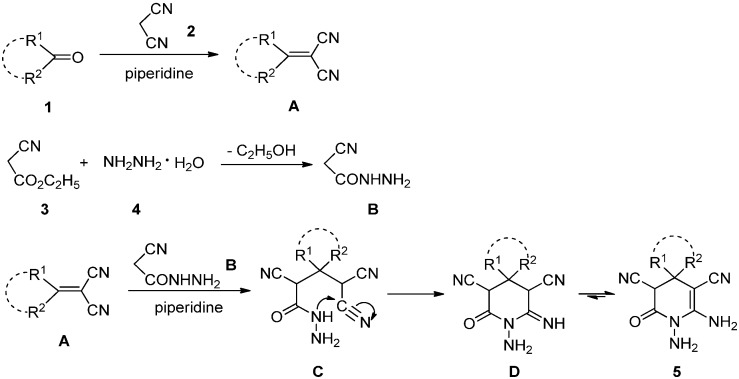
The proposed mechanism for the synthesis of compound **5**.

Although the detailed mechanism of this reaction has not yet been clarified, the formation of compounds **5** can be explained by the possible mechanism presented in Scheme 3. First, a Knoevenagel condensation of ketone **1** with malononitrile **2** is proposed to give the intermediate **A**. A condensation of ethyl cyanoacetate **3** with hydrazine hydrate **4** is also proposed to give the intermediate **B**. Michael addition of intermediate **B** to **A** catalyzed by piperidine should then occur to provide intermediate **C**, which undergoes intramolecular cyclization to give intermediate **D**. In the last step, the intermediate **D** is tautomerized to afford the product **5**.

## 3. Experimental

### 3.1. General Information

Melting points are uncorrected. IR spectra were recorded on Varian F-1000 spectrometer in KBr with absorptions in cm^−1^. All ^1^H-NMR data were determined on a Varian Inova-400 MHz but the ^13^C-NMR spectra had been run on the Varian Inova-300 MHz instrument, *J* values are in Hz. Chemical shifts are expressed in ppm downfield from internal standard TMS. HRMS analyses were carried out using a Bruker microTOF-Q instrument. X-ray diffraction was recorded on a Smart-1000 CCD diffractometer. Ultrasonication was performed in a KQ-250E medical ultrasound cleaner with a frequency of 40 kHz and an output power of 250 W. The reaction flask was located at the maximum energy area in the cleaner, and the surface of the reactions was placed slightly lower than the level of the water. Observation of the surface of the reaction solution during vertical adjustment of vessel depth will show the optimum position by the point at which maximum surface disturbance occurs. The reaction temperature was controlled by addition or removal of water from the ultrasonic bath.

### 3.2. General Procedure for the Synthesis of 1,6-Diamino-2-oxo-1,2,3,4-Tetrahydro-Pyridine-3,5-Dicarbonitrile Derivatives **5**

A 100 mL flask was charged with ketone **1** (1 mmol), malononitrile **2** (1 mmol), ethyl cyanoacetate **3** (1 mmol), hydrazine hydrate **4** (1 mmol) and piperidine (10 mol%, 0.1 mmol) in ethanol (10 mL). The mixture was sonicated in the water bath of an ultrasonic cleaner at 25–30 °C. After the completion of the reaction (monitored by TLC), the reaction mixture was concentrated in *vacuo* to remove the solvent. The residue was quenched with water and then filtered. The crude products were purified by recrystallization from ethanol to afford the pure products **5**.

*1,6-Diamino-4,4-dimethyl-2-oxo-1,2,3,4-tetrahydropyridine-3,5-dicarbonitrile* (**5a**). White solid, 147.7 mg, 72% yield; m.p.: 198–200 °C. IR (KBr, cm^−1^): 3415, 3346, 3305, 2175, 1702, 1632, 1569, 1428, 1340, 1218, 917, 858; ^1^H-NMR (400 MHz, DMSO-*d*_6_,): δ (ppm) 1.04 (s, 3H, CH_3_), 1.20 (s, 3H, CH_3_), 4.50 (s, 1H, CH), 5.15 (s, 2H, NH_2_), 6.61 (s, 2H, NH_2_). ^13^C-NMR (75 MHz, DMSO-*d*_6_): δ (ppm) 24.6, 27.0, 33.0, 47.9, 62.4, 115.8, 119.8, 153.8, 162.8; HRMS: calculated for C_9_H_10_N_5_O [M−H]^+^: 204.0885, found 204.0867.

*1,6-Diamino-4-methyl-2-oxo-4-propyl-1,2,3,4-tetrahydropyridine-3,5-dicarbonitrile* (**5b**). Black solid, 160.9 mg, 69% yield; m.p.: 136–138 °C. IR (KBr, cm^−^^1^): 3426, 3318, 2999, 2187, 1709, 1641, 1582, 1418, 1326, 1200, 942, 878, 785; ^1^H-NMR (400 MHz, DMSO-*d*_6_): δ (ppm) 0.85–0.86 (m, 3H, CH_3_), 1.10–1.27 (m, 7H, 2 × CH_2_,CH_3_), 4.65 (s, 1H, CH), 5.21 (s, 2H, NH_2_), 6.66 (s, 2H, NH_2_); ^13^C-NMR (75 MHz, DMSO-*d*_6_): δ (ppm) 13.7, 16.4, 23.9, 34.7, 43.9, 47.0, 59.1, 114.7, 119.5, 152.9, 162.0; HRMS: calculated for C_11_H_14_N_5_O [M−H]^+^: 232.1198, found 232.1186.

*1,6-Diamino-4,4-diethyl-2-oxo-1,2,3,4-tetrahydropyridine-3,5-dicarbonitrile* (**5c**). White solid, 163.3 mg, 70% yield; m.p.: 170–172 °C. IR (KBr, cm^−^^1^): 3445, 3317, 2988, 2195, 1707, 1645, 1576, 1418, 1369, 1206, 989, 886, 736; ^1^H-NMR (400 MHz, DMSO-*d*_6_): δ (ppm) 0.83 (t, *J* = 7.2 Hz, 3H, CH_3_), 0.91 (t, *J* = 7.2 Hz, 3H, CH_3_), 1.33–1.39 (m, 2H, CH_2_), 1.48–1.55 (m, 1H, CH), 1.78–1.82 (m, 1H, CH), 4.19 (s, 1H, CH), 5.20 (s, 2H, NH_2_), 6.72 (s, 2H, NH_2_); ^13^C-NMR (75 MHz, DMSO-*d*_6_): δ (ppm) 8.4, 8.9, 28.6, 30.8, 40.0, 43.7, 57.4, 115.4, 120.5, 155.0, 163.3; HRMS: calculated for C_11_H_14_N_5_O [M−H]^+^: 232.1198, found 232.1186.

*1,6-Diamino-4-cyclopropyl-4-methyl-2-oxo-1,2,3,4-tetrahydropyridine-3,5-dicarbonitrile* (**5d**). Gray solid, 138.6 mg, 60% yield; m.p.: 181–182 °C. IR (KBr, cm^−^^1^): 3624, 3446, 2880, 2189, 1713, 1637, 1565, 1431, 1284, 1224, 1166, 940, 839, 691; ^1^H-NMR (400 MHz, DMSO-*d*_6_): δ (ppm) −0.04–0.02 (m, 1H, CH), 0.10–0.16 (m, 1H, CH), 0.31–0.40 (m, 2H, CH_2_), 0.84–0.91 (m, 1H, CH), 1.32 (s, 3H, CH_3_), 4.72 (s, 1H, CH), 5.23 (s, 2H, NH_2_), 6.72 (s, 2H, NH_2_); ^13^C-NMR (75 MHz, DMSO-*d*_6_): δ (ppm) −0.7, 0.6, 16.1, 24.8, 34.8, 47.2, 54.9, 114.8, 119.6, 153.9, 161.9; HRMS: calculated for C_11_H_12_N_5_O [M−H]^+^: 230.1042, found 230.1039.

*2,3-Diamino-4-oxo-3-azaspiro[5.5]undec-1-ene-1,5-dicarbonitrile* (**5e**). White solid, 159.4 mg, 65% yield; m.p.: 156–158 °C. IR (KBr, cm^−^^1^): 3499, 3296, 2899, 2169, 1699, 1632, 1561, 1400, 1376, 1258, 914, 864, 681; ^1^H-NMR (400 MHz, DMSO-*d*_6_): δ (ppm) 1.04–1.07 (m, 1H, CH), 1.20–1.36 (m, 1H, CH), 1.45–1.70 (m, 8H, 4 × CH_2_), 4.52 (s, 1H, CH), 5.26 (s, 2H, NH_2_), 6.71 (s, 2H, NH_2_); ^13^C-NMR (75 MHz, DMSO-*d*_6_): δ (ppm) 21.4, 21.6, 25.3, 33.4, 34.2, 35.4, 45.8, 60.8, 115.9, 121.0, 154.8, 162.4; HRMS: calculated for C_12_H_14_N_5_O [M−H]^+^: 244.1198, found 244.1172.

*2,3-Diamino-9-methyl-4-oxo-3-azaspiro[5.5]undec-1-ene-1,5-dicarbonitrile* (**5f**). White solid, 173.6 mg, 67% yield; m.p.: 175–176 °C. IR (KBr, cm^−^^1^): 3426, 3318, 2164, 1709, 1626, 1538, 1410, 1299, 1148, 902, 836, 687; ^1^H-NMR (400 MHz, DMSO-*d*_6_): δ (ppm) 0.89 (d, *J* = 6.4 Hz, 3H, CH_3_), 1.14–1.22 (m, 1H, CH), 1.26–1.41 (m, 2H, CH_2_), 1.45–1.61 (m, 4H, 2 × CH_2_), 1.64–1.77 (m, 2H, CH_2_), 4.49 (s, 1H, CH), 5.23 (s, 2H, NH_2_), 6.68 (s, 2H, NH_2_); ^13^C-NMR (75 MHz, DMSO-*d*_6_): δ (ppm) 22.5, 30.4, 30.5, 31.6, 32.8, 34.1, 34.9, 48.8, 59.4, 115.8, 121.7, 155.2, 163.0; HRMS: calculated for C_13_H_16_N_5_O [M−H]^+^: 258.1335, found 258.1336.

*2,3-Diamino-9-ethyl-4-oxo-3-azaspiro[5.5]undec-1-ene-1,5-dicarbonitrile* (**5g**). White solid, 169.5 mg, 62% yield; m.p.: 184–186 °C. IR (KBr, cm^−^^1^): 3418, 3216, 2186, 1712, 1656, 1566, 1432, 1240, 1121, 956, 812, 662; ^1^H-NMR (400 MHz, DMSO-*d*_6_): δ (ppm) 0.86 (t, *J* = 6.4 Hz, 3H, CH_3_), 1.10–1.25 (m, 4H, 2 × CH_2_), 1.37 (t, *J* = 8.0 Hz, 1H, CH), 1.50–1.70 (m, 5H, 2 × CH_2_, CH), 1.77 (d, *J* = 9.6 Hz, 1H, CH), 4.50 (s, 1H, CH), 5.24 (s, 2H, NH_2_), 6.68 (s, 2H, NH_2_); ^13^C-NMR (75 MHz, DMSO-*d*_6_): δ (ppm) 11.7, 28.1, 29.4, 32.8, 34.1, 35.3, 38.2, 48.8, 59.4, 107.9, 115.8, 121.7, 155.2, 163.0; HRMS: calculated for C_14_H_18_N_5_O [M−H]^+^: 272.1511, found 272.1484.

*2,3-Diamino-9-benzyl-4-oxo-3,9-diazaspiro[5.5]undec-1-ene-1,5-dicarbonitrile* (**5h**). Yellow solid, 198.5 mg, 59% yield; m.p.: 114–116 °C. IR (KBr, cm^−^^1^): 3602, 3425, 3330, 2932, 2174, 1715, 1625, 1561, 1427, 1345, 1229, 1078, 985, 795, 740; ^1^H-NMR (400 MHz, DMSO-*d*_6_): δ (ppm) 1.68 (s, 4H, 2 × CH_2_), 2.28–2.60 (m, 4H, 2 × CH_2_), 3.48 (s, 2H, NCH_2_), 4.57 (s, 1H, CH), 5.25 (s, 2H, NH_2_), 6.78 (s, 2H, NH_2_), 7.25–7.35 (m, 5H, ArH); ^13^C-NMR (75 MHz, DMSO-*d*_6_): δ (ppm) 33.1, 33.7, 33.8, 46.3, 48.9, 49.2, 59.6, 62.5, 115.7, 121.2, 127.4, 128.6, 129.2, 138.8, 155.2, 162.5; HRMS: calculated for C_18_H_19_N_6_O [M−H]^+^: 335.1620, found 335.1619.

*tert-Butyl-8,9-diamino-7,11-dicyano-10-oxo-3,9-diazaspiro[5.5]undec-7-ene-3-carboxylate* (**5i**). White solid, 207.8 mg, 60% yield; m.p.: 222–224 °C. IR (KBr, cm^−^^1^): 3422, 3315, 3216, 2971, 2189, 1703, 1672, 1635, 1571, 1479, 1170, 897, 858, 769, 665; ^1^H-NMR (400 MHz, DMSO-*d*_6_): δ (ppm) 1.36–1.42 (m, 9H, C(CH_3_)_3_), 1.43–1.44 (m, 2H, CH_2_), 1.55–1.68 (m, 6H, 3 × CH_2_), 4.65 (s, 1H, CH), 5.24 (s, 2H, NH_2_), 6.86 (s, 2H, NH_2_); ^13^C-NMR (75 MHz, DMSO-*d*_6_): δ (ppm) 28.4, 32.7, 33.3, 33.9, 45.9, 58.6, 79.5, 115.5, 121.0, 154.3, 162.2; HRMS: calculated for C_16_H_21_N_6_O_3_ [M−H]^+^: 345.1675, found 345.1683.

*8,9-Diamino-10-oxo-3-thia-9-azaspiro[5.5]undec-7-ene-7,11-dicarbonitrile* (**5j**). Yellow solid, 158.0 mg, 60% yield; m.p.: 165–167 °C. IR (KBr, cm^−^^1^): 3427, 3340, 3041, 2926, 2179, 1725, 1633, 1552, 1430, 1349, 1064, 945, 829, 795; ^1^H-NMR (400 MHz, DMSO-*d*_6_): δ (ppm) 1.75–1.91 (m, 5H, 2 × CH_2 _and CH), 2.58–2.66 (m, 3H, CH_2_ and CH), 4.64 (s, 1H, CH), 5.20 (s, 2H, NH_2_), 6.18 (s, 2H, NH_2_); ^13^C-NMR (75 MHz, DMSO-*d*_6_): δ (ppm) 22.2, 22.3, 33.6, 33.7, 34.1, 44.6, 58.6, 114.6, 120.1, 154.1, 161.1; HRMS: calculated for C_11_H_12_N_5_OS [M−H]^+^: 262.0763, found 262.0741.

*2,3-Diamino-4-oxo-3-azaspiro[5.6]dodec-1-ene-1,5-dicarbonitrile* (**5k**). Gray solid, 165.9 mg, 64% yield; m.p.: 179–181 °C. IR (KBr, cm^−^^1^): 3356, 3189, 2198, 1703, 1686, 1589, 1453, 1256, 1141, 988, 852, 701; ^1^H-NMR (400 MHz, DMSO-*d*_6_): δ (ppm) 1.10 (s, 1H, CH), 1.26 (s, 1H, CH), 1.34–1.90 (m, 10H, 5 × CH_2_), 4.46 (s, 1H, CH), 5.24 (s, 2H, NH_2_), 6.67 (s, 2H, NH_2_); ^13^C-NMR (75 MHz, DMSO-*d*_6_): δ (ppm) 22.5, 23.0, 27.0, 30.3, 33.0, 35.8, 38.5, 47.8, 63.0, 116.3, 121.1, 154.2, 162.7; HRMS: calculated for C_13_H_16_N_5_O [M−H]^+^: 258.1355, found 258.1328.

## 4. Conclusions

In conclusion, we have described a novel approach to exploit the use of ultrasound irradiation for the synthesis of pyridin-2(*1H*)-one derivatives in ethanol solution at room temperature within 30–40 min. Compared with traditional methods, the procedure offers several advantages, including excellent yields, shorter reaction times, and a simple workup procedure.
